# Efficacy and toxicity of bevacizumab in recurrent ovarian disease: an update meta-analysis on phase III trials

**DOI:** 10.18632/oncotarget.6507

**Published:** 2015-12-08

**Authors:** Claudia Marchetti, Francesca De Felice, Innocenza Palaia, Angela Musella, Violante Di Donato, Maria Luisa Gasparri, Daniela Musio, Ludovico Muzii, Vincenzo Tombolini, Pierluigi Benedetti Panici

**Affiliations:** ^1^ Department of Obstetrics-Gynecological Sciences and Urological Sciences, Policlinico Umberto I “Sapienza” University of Rome, Viale del Policlinico, Rome 00161, Italy; ^2^ Department of Radiotherapy, Policlinico Umberto I “Sapienza” University of Rome, Viale Regina Elena, Rome 00161, Italy

**Keywords:** bevacizumab, ovarian cancer, recurrent, survival, toxicity

## Abstract

**Background:**

To analyze the efficacy and toxicity of bevacizumab on survival outcomes in recurrent ovarian cancer.

**Results:**

Bevacizumab was associated with significant improvement of PFS and OS compared with standard treatment with HRs of 0.53 (95% CI 0.44 − 0.63; *p* < 0.00001) and 0.87 (95% CI, 0.77 to 0.99; *p* = 0.03), respectively.

Bevacizumab increased the incidence of G3/G4 hypertension (RR 19.01, 95% CI 7.77 – 46.55; *p* < 0.00001), proteinuria (RR 17.31, 95% CI 5.42 − 55.25; *p* < 0.00001), arterial thromboembolic events (ATE) (RR 4.99, 95% CI 1.29 − 19.27; *p* = 0.02) and bleeding (RR 3.14, 95% CI 1.35 – 7.32; *p* = 0.008).

**Materials and Methods:**

Three randomized phase III trials representing 1502 patients were identified.

Pooled hazard ratio (HR), odd ratio (OR), risk ratio (RR) with 95% confidence interval (CI) were calculated using fixed or random effects model.

**Conclusions:**

Adding bevacizumab to standard chemotherapy improved ORR, PFS and OS, and it had a higher, but manageable, incidence of toxicities graded 3 to 4.

## INTRODUCTION

The vast majority of patients with primary epithelial ovarian cancer (OC) will experience a recurrence of their disease despite aggressive primary cytoreduction surgery and adjuvant cytotoxic chemotherapy.

Randomized phase III trials of bevacizumab in postoperative patients with primary OC have shown an improvement in progression free survival (PFS) without an appreciable significantly longer overall survival (OS) [1–2]. This benefit of bevacizumab incorporation into standard chemotherapy was also confirmed in recurrent disease after adjuvant platinum-based chemotherapy [3–4]. But neither of the two largest trials of bevacizumab in addition to standard chemotherapy in recurrent disease showed evidence of OS improvement over chemotherapy alone. Recently randomized GOG 213 trial has been presented and results have demonstrated improved PFS rates, as well as positive trend in OS, with HR 0.829 (95% CI 0.683 to 1.005, *p* = 0.056) [5]. However the survival benefit must be weighed in light of the acute toxicity. Thus we performed an update meta-analysis to include all randomized bevacizumab trials to test whether bevacizumab regimen in recurrent OC could be superior to standard chemotherapy, in term of efficacy and toxicity.

## RESULTS

### Description of patients

**Figure 1:**
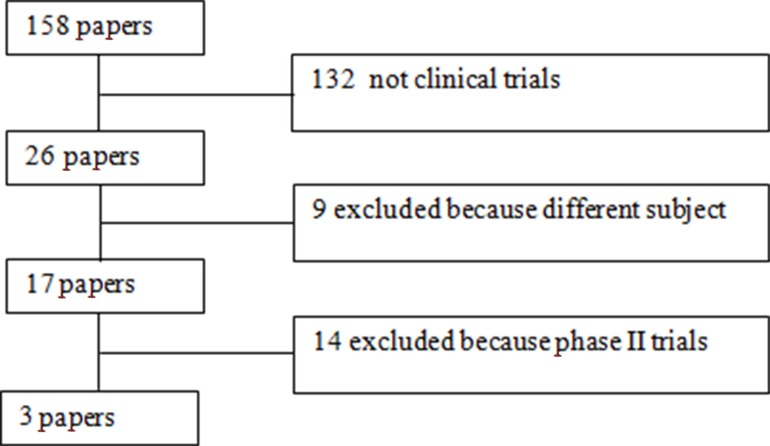
Flow-chart of meta-analysis

The selection of trials is depicted in the flow chart (Figure 1). Briefly, 158 articles were identified, of which 132 were excluded because they did not fulfill inclusion criteria. Twenty-six clinical trials were potentially eligible but 23 were excluded because they were not randomized phase III clinical trials. In total, 3 randomized phase III trials that evaluated bevacizumab plus chemotherapy versus chemotherapy alone for the treatment of recurrent OC were selected [3–5]. In the OCEANS trial no prior chemotherapy in the recurrent setting was allowed, whereas in the AURELIA trial and in the GOG 213 trial a total of 26 patients (7%) and 67 patients (10%), received prior antiangiogenic therapy, respectively.

### Overall survival and progression free survival

**Figure 2:**
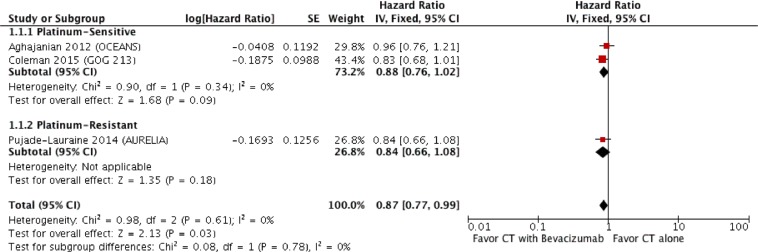
Forest plot for overall survival

The OS analysis was based on 3 trials, 1502 patients. The HR for OS was 0.87 (95% CI, 0.77 to 0.99; *p* = 0.03). There was no evidence of significant statistical heterogeneity with an *I*^2^ value of 0% (*χ*^2^ test for heterogeneity, *p* = 0.61). If we considered the only platinum-sensitive population, the HR became 0.88 (95% CI, 0.76 − 1.02; *p* = 0.09). The forest plot of OS is shown on Figure 2.

**Figure 3:**
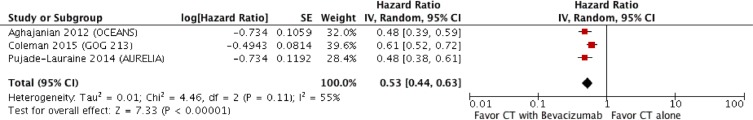
Forest plot for progression free survival

The benefit of bevacizumab on PFS was significant (HR 0.53, 95% CI 0.44 − 0.63; *p* < 0.00001; *χ*^2^ test for heterogeneity, *p* = 0.11; *I*^2^ = 55%; Figure 3).

### Objective response rate

**Figure 4:**
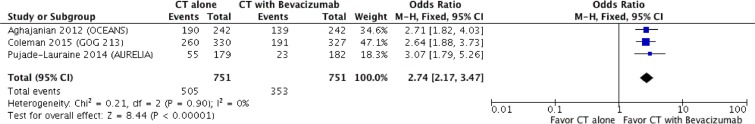
Forest plot for objective response rate

Bevacizumab has a significantly better ORR, with OR of 2.74 (95% CI 2.17 – 3.47; *p* < 0.00001; *χ*^2^ test for heterogeneity, *p* = 0.90; *I*^2^ = 0%). Details are shown in Figure 4.

### Toxicity

Among the 8 analyzed toxicities, only grade 3 to 4 hypertension (RR 19.01, 95% CI 7.77 – 46.55; *p* < 0.00001), proteinuria (RR 17.31, 95% CI 5.42 – 55.25; *p* < 0.00001), ATE (RR 4.99, 95% CI 1.29 – 19.27; *p* = 0.02) and bleeding (RR 3.14, 95% CI 1.35 – 7.32; *p* = 0.008) were significantly different between the two groups, with heterogeneity among trials. Data on the ATE were not available for OCEANS trial [3], thus ATE toxicity was calculated without those patients.

**Figure 5:**
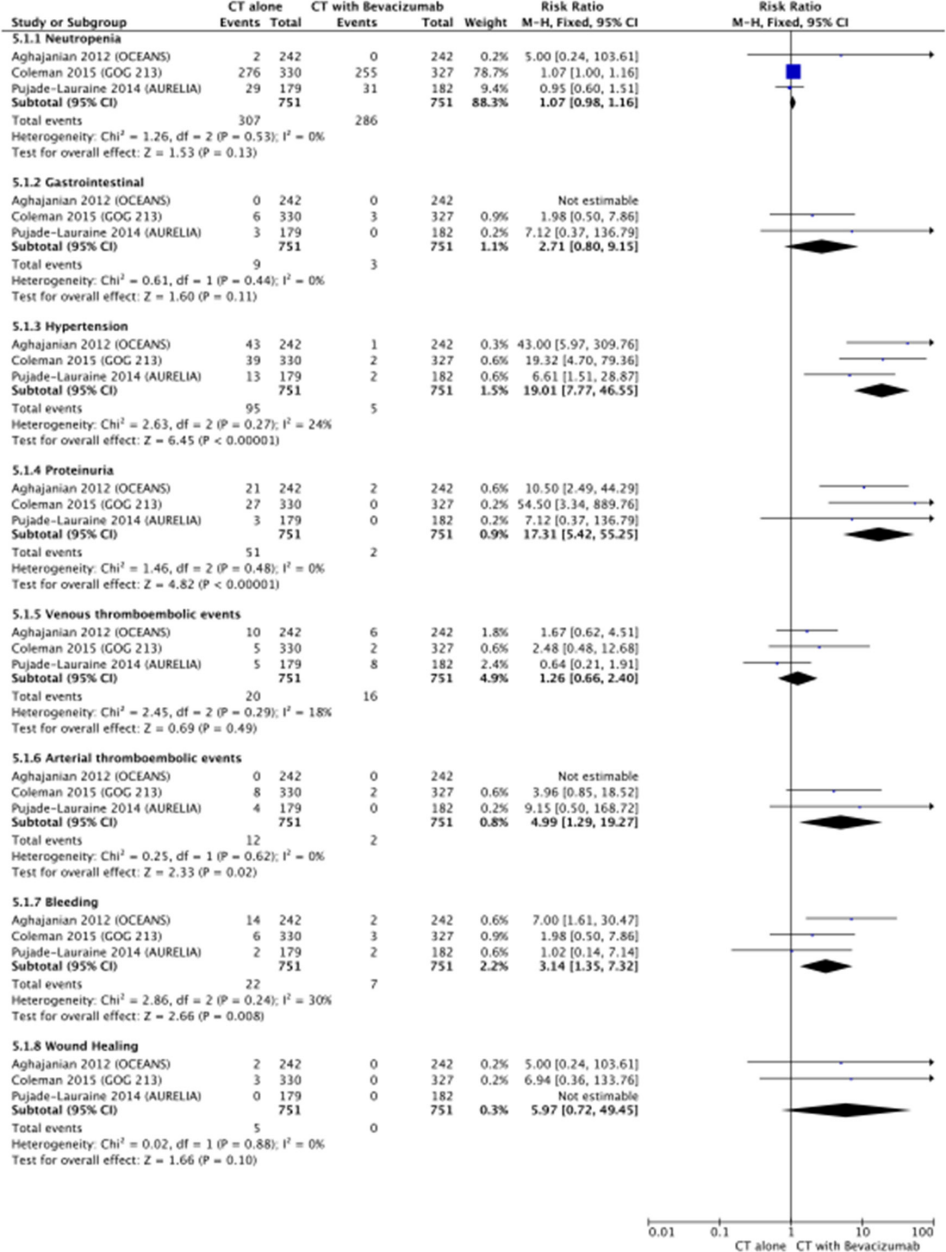
Forest plot for toxicity

Details of RR of toxicities associated with bevacizumab plus chemotherapy versus chemotherapy alone are shown in Figure 5.

## DISCUSSION

This meta-analysis provides an high level of evidence regarding the beneficial effect of bevacizumab in recurrent OC. The addiction of bevacizumab to standard chemotherapy confers a survival benefit, both progression-free and overall, which is consistent also in platinum-sensitive patients.

Actually, the VEGF-neutralizing monoclonal antibody bevacizumab has shown activity in OC treatment, on both first-line and recurrent setting and it has been the first anti-angiogenesis agent to be approved for treatment of OC in the front-line setting [1–2]. In the recurrent setting, two large randomized clinical trials have demonstrated the benefit of the addition of bevacizumab to a second-line regimen in both platinum sensitive [3] and platinum resistant [4] settings. No OS benefit was found, in both trials. More recently the GOG 213 trial [5], found that paclitaxel, carboplatin, and bevacizumab extended OS in patients with platinum-sensitive recurrent OC, but narrowly missed that statistical upper limit of significance. The combination was also associated with a significant improvement in PFS as well as ORR.

This meta-analysis consists of OC patients with recurrent disease included in randomized trials of bevacizumab combined with chemotherapy. In the light of the most recent evidences and with the update of each trial, it adds significant evidences compared with previous published meta-analysis [6–7].

Our analysis showed that the combination of bevacizumab with standard treatment of recurrent OC is beneficial in prolonging PFS (HR 0.53, 95% CI 0.44 − 0.63) and assuring an increased response rate (OR 2.74, 95% CI 2.17 – 3.47). Unfortunately, we did not analyze data concerning QoL as they were lacking or not homogeneous. Active treatment is generally undertaken with the goals of providing improved quantity and/or quality of patient survival. It has been demonstrated a strong association between PFS, cancer-related symptoms, and QoL among patients with cancer [8]. Nonetheless, it should be underlined that in both the AURELIA trials and in the GOG 213 trial a quality-of-life assessment showed no deterioration of life quality in patients randomized to bevacizumab. Notably, platinum-resistant OC patients form the AURELIA trial receiving bevacizumab plus chemotherapy showed a response rate of more than 30%, and significant prolongation of PFS but also a 15% improvement in abdominal and gastrointestinal symptoms, significantly greater than in the chemotherapy group (21.9% versus 9.3%, 95% CI: 4.4 – 20.9, *p* = 0.002) [9]. Therefore it might be speculate that the adjunct of bevacizumab has not any detrimental effect in terms of QoL; conversely, it might have a positive effect in those with the greatest symptomatology, as the platinum resistant group of patients.

In terms of toxicity, we have analyzed G3/G4 toxicities as we focused on those toxicities that might have been disadvantageous in terms of outcomes and QoL. Overall, there were more side effects in the group that used bevacizumab-containing regimen but all remained within expected parameters and also toxicities were all manageable. Interestingly, should be noticed that toxicity data from the GOG 213, which included approximately 10% of women who had previously received bevacizumab, are in agreement with those previously published, with an higher occurrence of G3/G4 thromboembolisms, hypertension and proteinuria compared with standard arm. As there is an increase in toxicity, attention should be given to patients that have an increased risk of bleeding, recent or current use of aspirin or oral and/or parenteral anticoagulants. Hypertension and proteinuria are usually controllable events and do not require permanent discontinuation of bevacizumab.

Finally, we also presented data about OS, which were not mature when previous meta-analysis have been published. The addition of bevacizumab was associated with a small but significant improvement in OS (HR:0.87; 95% CI 0.77 − 0.99). When considering only platinum sensitive patients, the analysis points again to a benefit of chemotherapy plus bevacizuamb, although in a different extent (HR 0.88, 95% CI, 0.76 − 1.02).

Remarkably, it should be pointed out that AURELIA and OCEANS trials were designed and powered to evaluate PFS and not OS as primary end-point; conversely, GOG 213 trial had OS as primary end-point and narrowly missed the significance (*p* = 0.056). Nonetheless, when it was designed in 2007 the superiority of adding the angiogenesis inhibitor was not still proven and therefore investigators used two-tailed statistical analysis. But currently, as bevacizuamb's knowledge has increased, it is more common to use a one-tailed test, which would have allowed the significance to be reached. Moreover, the estimated median OS of control arm was fixed to 22 months and this underestimation might have contribute to the trial to miss the statistical cut-off.

This trend was found also when considering only sensitive disease but without clear significance, suggesting that further prospective studies are needed to investigate if OS could be improved through bevacizumab plus standard chemotherapy in some selected population.

Furthermore, even if no definitive evaluation of the usefulness of bevacizumab beyond chemotherapy can be made within the current meta-analysis, should be underlined that in all 3 studies, differently from randomized studies in the first-line setting in which no global OS benefit was found [1–2], bevacizumab was administered as monotherapy until disease progression or unacceptable toxicity in those patients who did not progress during the protocol of the six cycles of combination. This might be of interest in the debate concerning the length of administration of this compound, which is the object of several trials (BOOST trial, NCT01462890; MITO16/MANGO2b trial; NCT01802749) currently ongoing in the first line setting.

Our analysis was limited by its use of summary data rather than data from the individual patients from each trial. Individual patient data are needed to better account for the control arm, to standardize the analysis to perform an intent-to-treat analysis, to draw survival curves, to perform a more complete analysis of the variation of treatment effects according to patient. It was originally intended that this summary level analysis would be followed by an analysis of individual patient data from the eligible trials but, ultimately, this has not been possible.

Further trials are needed, which should also consider the QoL as well as the cost of bevacizumab plus chemotherapy combination in the recurrent setting; moreover the next challenge is to identify those biomarkers that might allow to better define the subgroup population who may benefit at most of this compound.

## MATERIALS AND METHODS

### Data collection and trials selection

The Preferred Reporting Items for Systematic Reviews and Meta-Analyses (PRISMA) statement was followed to perform the meta-analysis. It includes randomized clinical trials, written in English, without any restrictions on publication date. The last search was done on July 2015. Literature electronic databases (Pubmed, Medline and Scopus) were searched for “recurrent”, “ovarian cancer” and “bevacizumab” in the title. Trials that compared bevacizumab plus chemotherapy administration to standard chemotherapy alone in women with recurrent OC were eligible. To reduce publication bias, data from all clinical randomized trials, both published and unpublished, were included using literature electronic databases searching (Pubmed, Medline and Scopus) and hand searching (meeting proceedings of Society of Gynecologic Oncology, European Society of Medical Oncology and American Society of Clinical Oncology). Reference lists of previously published reviews and meta-analyses were explored. Review articles, case reports, commentaries and letters were not included.

Two independent investigators (CM and FDF) selected the identified studies based on the title and abstract. If the study's topic could not be ascertained from its title or abstract, the full-text version would be retrieved for evaluation. Disagreement was resolved by discussion or consensus or with a third party (LM).

Trials were eligible if patients had a proven OC recurrence. In the closer evaluation of potentially eligible articles, when two articles appeared to report results with overlapping data, only the data representing the most recent publication date were included in the meta-analysis. From all including studies were extracted: first author's last name, publication year, the study name, sample size of cases and controls, regimen used, data on PFS, OS, objective response rate (ORR) and acute toxicities ≥ G3. Update information on survival and date of last follow-up were requested.

### End-points

End-points were the PFS, defined as the time from random assignment to progression disease or death, the OS, defined as the time from randomization to death, the objective response rate (ORR) and toxicity. The hazard ratios (HR) and 95% confidence interval (CI) for PFS and OS were derived from each study; whereas for ORR and toxicities were derived the odd ratios (OR) and risk ratios (RR), respectively.

The toxicities analyzed were graded > 2 and were gastrointestinal (GI), hypertension, proteinuria, venous thromboembolic events (VTE), arterial thromboembolic events (ATE), bleeding, would healing and neutropenia. If the grade ≥ 3 adverse events were not directly provided in the text, they were estimated resulting from data in the appropriate Figure/Table.

### Statistical analysis

Statistical analysis of pooled ORR, PFS, OS and toxicities were performed using Review manager 5.0 software (http://www.cochrane.org). The pooled HR, OR and RR were calculated using a fixed or random effect models, depending on heterogeneity. Forest plot were used for graphical representation of each study and pooled analysis.

The size of every box represents the weight that the corresponding study exerts in the meta- analysis; CI of each study are displayed as horizontal line through the box. The pooled HR, OR and RR are symbolized by a solid diamond at the bottom of the forest plot and the width of the square represents the 95% CI of HR.

HR, OR, RR and 95% CI for each study were extracted or calculated based on the published studies according to the methods described by Tierney in 2007 [10]. A significant two-way *p*-value for comparison was defined as *p* < 0.05. The size of the square represents the weight that the corresponding study exerts in the meta-analysis. Statistical heterogeneity between studies was examined using both the Cochrane Q statistic (significant at *p* < 0.1) and the *I*^2^ value (significant heterogeneity if > 50%) [11]. Publication bias was examined using analyses described by Egger and Begg [12–13].

## CONCLUSION

This meta-analysis of randomized studies indicates that integrate bevacizumab in the standard treatment for patients with recurrent OC prolongs PFS and OS, without unexpected toxicity patterns.
